# SyNDI: synchronous network data integration framework

**DOI:** 10.1186/s12859-018-2426-5

**Published:** 2018-11-06

**Authors:** Erno Lindfors, Jesse C. J. van Dam, Carolyn Ming Chi Lam, Niels A. Zondervan, Vitor A. P. Martins dos Santos, Maria Suarez-Diez

**Affiliations:** 1 LifeGlimmer GmbH, Markelstrasse 38, 12163 Berlin, Germany; 2 Laboratory of Systems and Synthetic Biology, Wageningen University & Research, Stippeneng 4, 6708 WE Wageningen, The Netherlands

**Keywords:** Synchronous network visualization, Workflow, Cytoscape, Galaxy, Network biology, Systems biology, *Mycobacterium tuberculosis*, *Staphylococcus aureus*

## Abstract

**Background:**

Systems biology takes a holistic approach by handling biomolecules and their interactions as big systems. Network based approach has emerged as a natural way to model these systems with the idea of representing biomolecules as nodes and their interactions as edges. Very often the input data come from various sorts of omics analyses. Those resulting networks sometimes describe a wide range of aspects, for example different experiment conditions, species, tissue types, stimulating factors, mutants, or simply distinct interaction features of the same network produced by different algorithms. For these scenarios, synchronous visualization of more than one distinct network is an excellent mean to explore all the relevant networks efficiently. In addition, complementary analysis methods are needed and they should work in a workflow manner in order to gain maximal biological insights.

**Results:**

In order to address the aforementioned needs, we have developed a Synchronous Network Data Integration (SyNDI) framework. This framework contains SyncVis, a Cytoscape application for user-friendly synchronous and simultaneous visualization of multiple biological networks, and it is seamlessly integrated with other bioinformatics tools via the Galaxy platform. We demonstrated the functionality and usability of the framework with three biological examples - we analyzed the distinct connectivity of plasma metabolites in networks associated with high or low latent cardiovascular disease risk; deeper insights were obtained from a few similar inflammatory response pathways in *Staphylococcus aureus* infection common to human and mouse; and regulatory motifs which have not been reported associated with transcriptional adaptations of *Mycobacterium tuberculosis* were identified.

**Conclusions:**

Our SyNDI framework couples synchronous network visualization seamlessly with additional bioinformatics tools. The user can easily tailor the framework for his/her needs by adding new tools and datasets to the Galaxy platform.

**Electronic supplementary material:**

The online version of this article (10.1186/s12859-018-2426-5) contains supplementary material, which is available to authorized users.

## Background

Systems biology promotes a holistic approach in which biological elements such as molecules or reactions are no longer considered in isolation but as components of a bigger system such as a cell [1]. Within this framework, networks provide a natural way to describe associations and interconnections between system components. Network biology has emerged as one of the core sub-fields of systems biology in which nodes are biomolecules (e.g. proteins, genes, and metabolites) and edges represent interactions, associations and relationships between the biomolecules (e.g. chemical conversions, signal transduction steps, regulations, and co-expressions) [2] This approach is creating new inroads to solutions and applications in systems medicine [3] and industrial biotechnology [4] among others.

The reconstructed networks are usually mined using a variety of querying methods [3–5]. In many cases, these methods aim at selection of sub-networks based on experimental evidence or on local topological properties (e.g. identification of network clusters) [6]. Computational analysis methods are in turn applied on selected sub-networks to understand related biological context. For example, Gene Ontology (GO) enrichment analysis can be performed to associate sets of genes or proteins with a specific biological process [7] or motif identification in upstream regions of selected genes [8] to identify gene regulators.

Biological network visualization has remained a highly non-trivial task and one of those currently open challenges related to the need of simultaneous network visualization to optimally and efficiently perform differential network analysis. Many alternative methods can be used to extract networks from the same datasets and the resulting networks have to be examined to generate a consensus network [9]. Different network representations are needed to convey different layers of information pertaining the same system (e.g. metabolic networks, protein-protein interactions networks, gene regulation networks), however these information layers are not independent and all of them have to be considered as a whole in order to describe how the overall system behaves. Moreover, different networks might arise even when considering similar biological processes under different conditions (e.g. healthy versus disease states) [10].

As a result of this multiplicity in the nature of networks and the subsequent integration need, many advanced graph-based methods have been developed for comparing networks [10]. Some of them produce local measures for individual nodes (e.g. node degrees, clustering coefficients) and these are compared on a node basis across different networks. This can, in turn, help clarifying the biological significance of a highly connected node, or hub. Other methods give global measures for the network as a whole, for instance distributions and average values for node degree and clustering coefficients, and network diameter [11]. A researcher is needed to interactively inspect these results to achieve proper analysis and interpretation.

As stated, network analysis requires the use of complementary analysis methods. In today’s omics era it has become utmost important that these data analyses can be performed through the use of consistent workflows, where results can be stored for further analysis and findings can be reliably reproduced. In addition, these workflows have to be integrated with network visualizations, so that it is possible to easily switch from network interpretation to subsequent bioinformatics data analysis and vice versa. Galaxy is a user-friendly web-based platform that has been developed to address these needs [12–14].

Here we present SyNDI, a Synchronous Network Data Integration framework for synchronous visualization of multiple biological networks that addresses the above mentioned challenges. Specifically, the SyNDI framework endows Cytoscape [15] with the capability to show multiple networks in a synchronous way that preserves the identity between nodes appearing in multiple networks, thus enabling visually inspecting differences in their local connections. SyNDI also provides the possibility to perform data analysis directly from the network visualization (without complicated file handlings) using Galaxy and vice versa - the analysis results from Galaxy can be directly exported to the network visualization.

Here we demonstrated the functionality and usability of SyNDI with three biological examples. First, we illustrated how it can be used to assist analysis of metabolite association networks related to high and low latent cardiovascular risk respectively by simultaneously visualizing those networks. In our second example, we analyzed a few common response pathways between human and mice in *Staphylococcus aureus* infection to gain further biological insights. Finally, we demonstrated how SyNDI connects network visualization with Galaxy’s data analysis tools and specifically we analysed type VII secretion system, ESX-1, in a human pathogen *Mycobacterium tuberculosis*; this study represents a follow-up on an earlier analysis of key regulatory events associated with pathogenesis and survival within the host [16].

## Implementation

**Fig. 1:**
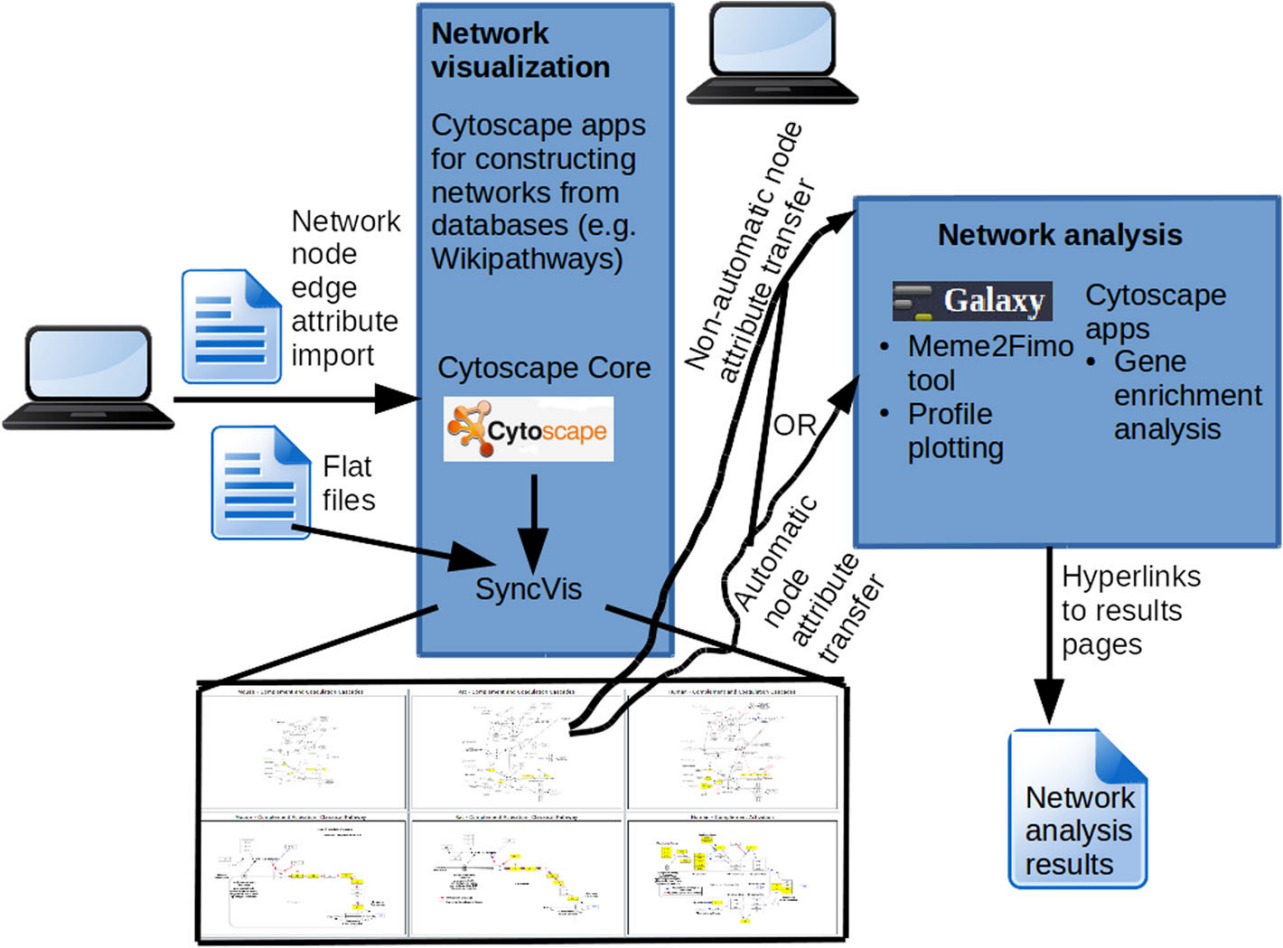
Technical architecture of a workflow system. It comprises of layers for network visualization and analysis; synchronous network visualization on a SyncVis Cytoscape app and network analysis on Galaxy or another external tool. The user can transfer node attributes from SyncVis to a network analysis to automatically or non-automatically

The overall architecture of our framework is presented in Fig. 1. It is composed of two layers:

- SyncVis is a Cytospape app that allows the user to visualize multiple biological networks exploiting the Cytoscape core.
- Network analysis layer which uses Galaxy [2–4] for central core of analysis.

In the next sub-sections we describe these layers in technical detail.

### Network visualization

We have developed a Cytoscape app called SyncVis (Synchronous Visualizer) for network visualization. Also we use Cytoscape core for some of this functionality. In the next sub-sections we describe the technical implementation in detail. In the first sub-section we present a few options the user can apply for constructing networks as a pre-step before starting to use the SyNDI framework. Then we describe three other sub-functionalities: Network import, synchronous visualization and node attribute export.

#### Pre-step - network construction

In order to visualize networks on the SyNDI network, the user needs to construct networks. We would like to emphasize this procedure is not part of SyNDI framework. However we feel this procedure deserves its own sub-section since it is a necessary pre-step - the user needs to have sufficient knowledge about network construction in order to use the SyNDI framework.

She can use a top-down approach to generate networks from experimental data using existing reconstruction algorithms [13]. In most cases it is pragmatic to implement these algorithms as separate applications for example in the R environment.

Alternatively the user can use a bottom-up approach by constructing networks from available biological databases (e.g. signaling pathway databases, metabolic pathway databases, protein-protein interaction databases). Most of biological pathways have some networks directly available on their web sites - for example Wikipathways database [17, 18] has hundreds of pathways available (http://www.wikipathways.org). In addition, these pathways usually have Application Programming Interface (API) available that support high-level programming languages (e.g. Java). The user can use these APIs to implement an application customized for her purpose. Some of these databases have been integrated in common bioinformatics tools - for example Wikipathways database has a Cytoscape app (http://apps.cytoscape.org/apps/wikipathways) that the user can use to retrieve pathways based on various search parameters directly on Cytoscape.

#### Network import

Cytoscape core supports most of the base network representation formats like Simple Interaction Format (SIF), eXtensible Graph Markup and Modeling Language (XGMML) and Systems Biology Markup Language (SBML). Most of the networks construction tools and methods covered in the previous sub-section can generate networks in some of these formats. The user can therefore import networks to Cytoscape Core for example by using “ctrl + L” shortcut key or “File -> Import -> Network” menu. In some case the user may have additional parameters for nodes or edges in separate files. This can be the case for example if she has used a separate tool to calculate log2 fold changes and statistical metrics like *p*-values from transcriptomics data. Technically this happens by using the “File -> Import -> Table” menu on Cytoscape core.

Alternatively the user can use a specific Cytoscape app like the Wikipathways Cytoscape app mentioned in the previous sub-section to construct networks directly on Cytoscape.

#### Synchronous visualization

The concept of synchronous network visualization is illustrated on the bottom of Fig. 1. Typically the user goes though the following pipeline when using this feature.

- The user has a specific node of interest (e.g. an individual gene) or a group of nodes (e.g. genes involved in a specific biological process).
- The user search the node(s) on one network (e.g. on an organism specific pathway)
- The corresponding node(s) are automatically highlighted on another network (e.g. on a similar pathway from another organism). The user can thus easily look into the differences in local connections of the nodes between the networks.

The same pipeline can be applied to a synchronous visualization of any other networks (e.g. networks from different medical conditions, networks produced by different network construction algorithms).

We have implemented a Cytoscape app called SyncVis (Synchronous Visualizer) for this functionality by using Cytoscape Java API package (http://chianti.ucsd.edu/cytoscape-3.5.1/API/). We map the node selections via Cytoscape’s “shared name” attribute which means that node identifies (e.g. gene names) have to be stored in this attribute. Next we will present simplified code snippets demonstrating how these mappings are implemented on Java programming level.

// First we retrieve selected nodes from Cytocape’s “selected” attribute: // selCyNet the network on which the user selects the nodes.

List<CyNode> selNodes = CyTableUtil.getNodesInState(selCyNet,“selected”,true); // Then we store the “shared name” attributes of the selected nodes in a hash set: HashSet<String> selSharedNames = new HashSet<String>(); for (CyNode node: selNodes) { String sharedName = cyNodeTable.getRow(node.getSUID()).get(“shared name”,String.class); selSharedNames.add(sharedName); }

// Then we select the nodes of the other networks based on their presence in selSharedNames: // allNets is a list that contains all networks that are imported in Cytoscape for (CyNetwork cyNet: allNets) { CyTable cyNodeTable = selCyNet.getDefaultNodeTable(); for (CyNode node: selCyNet.getNodeList()) { CyRow row = cyNodeTable.getRow(node.getSUID()); String sharedName = row.get(“shared name”,String.class); row.set(“selected”, selSharedNames.contains(sharedName)); }}

In addition the user can upload his/her own mapping file (e.g. homologs between two species). We have explained this procedure in the user manual.

#### Node attribute export

SyncVis needs functionalities to export node attribute data for smooth communication with network analysis. Cytoscape’s “shared name” attribute is used to for this connection and it is accessed in the same way on Java programming level as in synchronous visualization as described in the previous sub-section.

As indicated in Fig. 1, SyncVis contains two alternative options for the export:

- *Automatic export*
  In this option the node data transfer from SyncVis to the Galaxy platform is automated; SyncVis communicates automatically with Galaxy and the user does not need do any manual operation. SyncVis contains two buttons to this operation for each network analysis: one button that creates a flat file when the user clicks on it and another button that sends the request the Galaxy platform when the user clicks on it and has possibly given additional parameters for network analysis. Technically this is implemented so that first SyncVis creates a flat file that contains the “shared name” attributes of the selected nodes. Then it calls a python script from Java code that uses a BioBlend API [19] to send the flat file to the Galaxy platform as an input of network analysis.
- *Non-automatic export*
  In this option user interventions is needed for the node data transfer from SyncVis to the Galaxy platform (or another network analysis tool such as a BiNGO Cytocape app [20]). First a flat file is created. This is done manually and using a button on SyncVis to copy-paste the “shared name” attributes of the selected nodes to the flat file. Alternatively the user can click another button on SyncVis to save the attributes to a flat file. The flat files can then be imported into Galaxy or another network analysis tool.

SyncVis has these two alternative options in order to find a balance between automated communication and software development. The automated export option is a very user-friendly but some technical work is needed to implement it on SyncVis for a network analysis tool. For time being this export is therefore implemented only for a few network analysis tool. The non-automatic export is not so user friendly but this is does not require any extra work from the software developer, so the user can use it immediately if she wants use a network analysis tool for which the automatic export is not implemented.

**Fig. 2:**
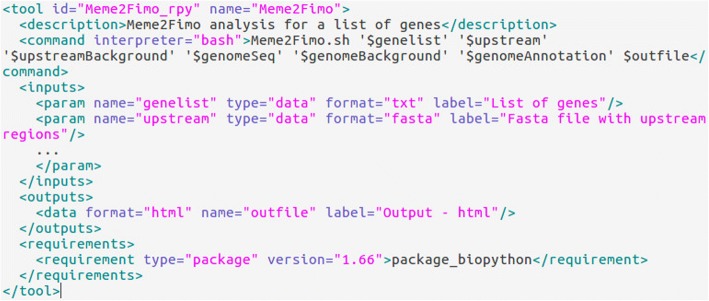
Content of an XML file that defines a Galaxy tool. This file contains a brief description of the tool, a command for running the tool, and the input and output parameters of the tool

Figure 2 illustrates the content of an Extensible Markup Language (XML) file defining a connection between SyncVis and a tool running on the Galaxy platform. The command element defines how the Galaxy platform executes the tool using the input files listed in the input element. The output element defines the format of the response. Algorithms can be implemented by any programming language that Galaxy supports (e.g. R, Python, bash). More details about the content of this file can be found at the tool configuration page at the wiki page of the Galaxy project (https://docs.galaxyproject.org/en/latest/dev/schema.html).

SyncVis needs an API key for the connection with the Galaxy platform. Our user manual contains detailed instructions for configuring this key.

### Network analysis

The purpose of network analysis is to gain our understanding of the underlying biology behind a visualized network. The user can select sets of genes for further investigation on the visualized network. She can for example perform a Gene ontology (GO) enrichment analysis to see in what biological processes the genes are over-represented, or plot the gene expression profiles or search shared sequence motifs between the genes.

We use a Galaxy as a central platform for running these analyses since it is a widely used platform for running bioinformatics analysis requiring no programming skills from end users. When the Galaxy platform has completed analysis, it reports the results on Hyper Text Markup Language (HTML) pages to which SyncVis displays links on pop-up windows.

The SyNDI framework is not restricted to Galaxy as it can easily interoperate with other available analysis tools and Cytoscape applications, such as Biological Networks Gene Ontology tool (BiNGO) [20].

### Meme2Fimo tool

We have implemented a tool called Meme2Fimo in the Galaxy server for upstream sequence analysis. Meme2Fimo integrates tools for motif identification (MEME [12]) and motif search (FIMO [39]).

From a user given gene selection, MEME is used to identify an up to 5 possible motifs in the upstream regions of the selected genes, which are automatically retrieved from a GenBank file. MEME is executed with the “-dna -revcomp -nmotifs 5 -mod zoops -evt 1000” parameter string. MEME generates a list of found motifs and for each motif it returns an ordered list of scores for the selected input genes. The score indicates how well the motif fits to the identified upstream region. These motifs and associated ordered list are collected and stored.

For each motif identified by MEME, FIMO is executed to locate any other occurrences within the complete genome. FIMO is executed with the “--bgfile <genome background>” option. The genome background is generated from the complete genome sequence using the “fasta-get-markov” command, with an order value of 3. FIMO returns a list of occurences with an associated location, *p*-value and q-value. This list is ordered by *p*-values. All occurrences that occur within a known gene are rejected. For each remaining occurrence, Meme2Fimo searches for the gene downstream and upstream if present. If for the given downstream gene already another occurrence is found, then it is rejected and the hit count of the already found occurence is increased by one. If the downstream gene is present within the stored list captured from the MEME output, the index within that list is added to final output of Meme2Fimo. Otherwise a − 1 is added. So Meme2Fimo will add for each motif result generated by MEME to an additional table, which contains a row for each accepted occurrence found in the genome of that motif: a downstream gene identifier, a sequence associated to the occurrence, an index of the downstream gene within the initial MEME result, an index of the upstream gene within the initial MEME result, a *p*-value, a q-value, a hit count and relative position to the downstream gene.

Based on the index values one can identify other genes that are regulated by the same regulator. If in the top hits within the list some occurrences and associated genes are found, which are not within the selected set of genes (indicated with a − 1) one can add these genes to the input and rerun Meme2Fimo. If one keeps repeating this process, in some cases (e.g. in a motif related to the DosR regulator presented in Results and Discussion) the indexes in the list will converge to a list without any − 1 values in between.

## Results and discussion

### Probabilistic networks of blood metabolites associated to latent cardiovascular risk

Comparison of networks extracted under different clinical conditions, such as health and disease, might help uncover key mechanisms of disease physiology, especially in conditions whose outcome is presumably affected by a multitude of risk factors. Cardiovascular diseases (CVD), one of the leading causes of death in western countries, are associated to risk factors of metabolic origin, however the complex nature of CVD has prevented a complete mechanistic understanding of these risk factors and their associations.

In a previous study [21], a global analysis was performed on the association networks between a panel of metabolites quantified using Nuclear Magnetic Resonance (NMR) from plasma samples from healthy individuals. Metabolites’ association networks were defined for individuals with low CVD risk and for those presenting latent CVD risk. Briefly, an array of 29 metabolites identified and quantified in the plasma of 864 healthy blood donors of both genders was considered [22]. Clinical data and traits: concentrations of high and low density lipoproteins (HDL and LDL respectively), total cholesterol, triglycerides, glycaemia and Framingham score, were used to split the cohort according to latent CVD risk levels: low, medium or high. Metabolite networks associated to high and low CVD latent risk were extracted using the Probabilistic Context Likelihood of Relatedness based on Correlation (PCLRC) algorithm [21].

**Fig. 3:**
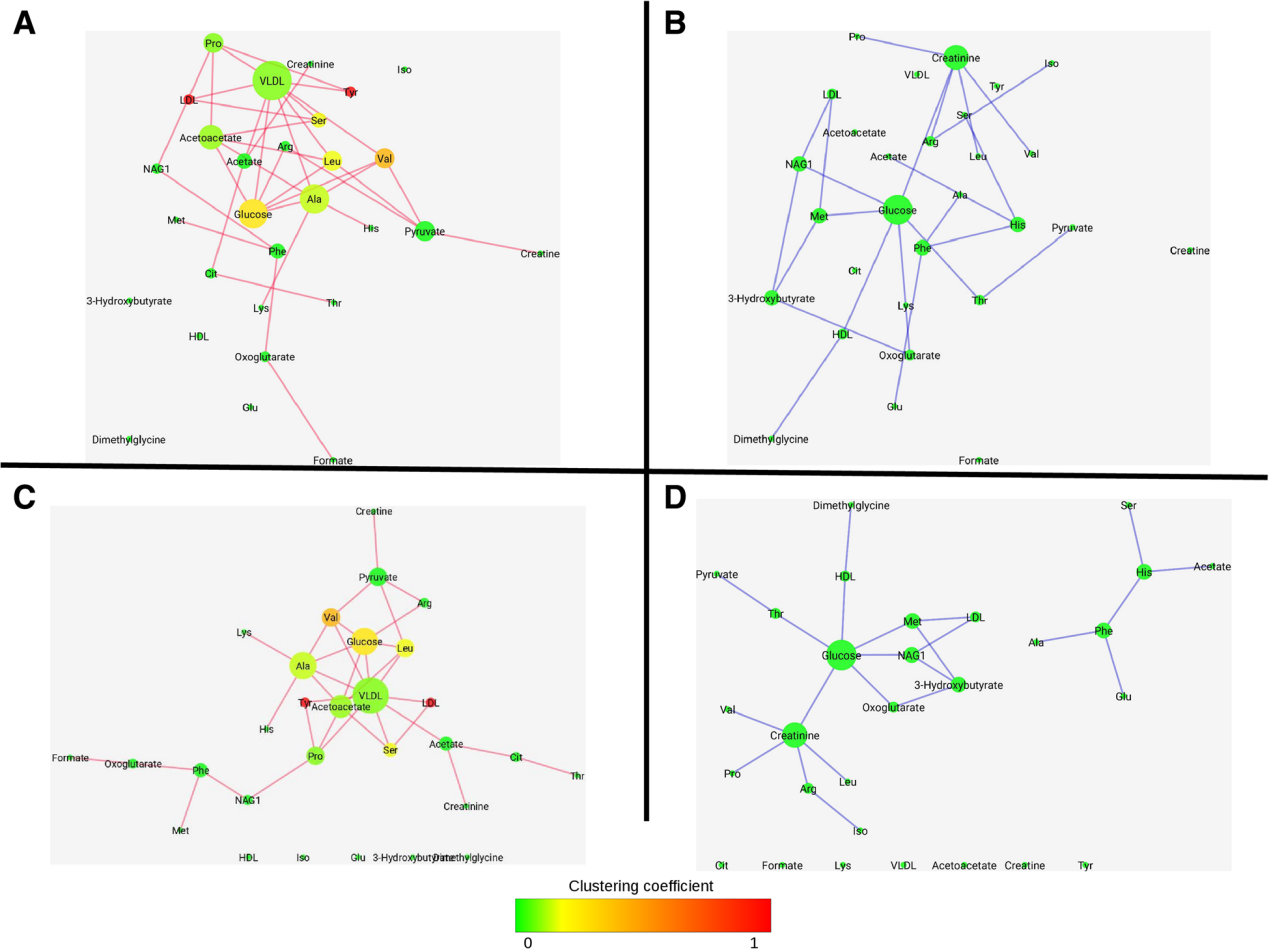
Association networks of blood metabolites. Nodes represent metabolites. Node size is proportional to node degree and node color is linked to clustering coefficient. **a** and **c**: Associations found exclusively in subjects with high latent CVD risk (red edges). **b** and **d**: Associations found exclusively in subjects with low latent CVD risk (blue edges). Networks in A and B have the same node location. Networks C and D have been obtained using force directed layout in each of them

Figure 3 represents the associations linked to either high (panels A and C) or low (panels B and D) latent CVD. Topological indices for each node, such as clustering coefficient and degree are represented by node color and size respectively. Using a common layout for both networks eases the comparison, as nodes occupy the same relative position in both networks (compare panels A and B of Fig. 3). However, a dedicated layout for each of them, (Fig. 3c and d) eases the identification of the key local connections. These network representations emphasize, for instance, the prominent location of very-low-density lipoprotein (VLDL) in the high latent risk network (Fig. 3c) or the two connected components in the low CVD risk network (Fig. 3d) that highlights the association between acetate and the amino acids serine, histidine, phenylalanine, glutamine and alanine. In the high latent risk network these latter associations are disrupted and glucose appears associated to amino acids, which are known mediators of glucose metabolism, insulin secretion, and insulin sensitivity [23].

However, these networks pe se are not enough for a smooth local view switch network. SyncVis tackles this challenge by transferring node selections between networks automatically.

### Synchronous visualization of differentially expressed genes under *S. aureus* infection on human and mouse signaling pathways

In order to demonstrate SyNDI’s functionality for synchronous network visualization of networks across different species, we visualized Differentially Expressed (DE) genes in the context of *S. aureus* infection in human and mouse. We thus aimed to gain deeper insights among dysregulated pathways shared by these two species during *S. aureus* infection. Banchereau et al. performed whole transcriptomics analysis on *S. aureus* infected patients and healthy people (99 and 44 samples respectively) [24]. This data set comprises 24,371 transcripts. DE genes were identified (False Discovery Rate (FDR) < 0.01 and log2 fold change > 0.7). Brady et al. studied protective mechanisms in mice to *S. aureus* Skin and Soft Tissue Infection (SSTI) [25]. They used an SSTI mouse model to study local (=infected vs non-infected ears) and systemic (=challenged vs naïve mice) responses to infection at one, four and seven days after the start of infection. RNA sequencing (RNA-seq) was used and DE genes were defined as those with a log2 fold change of 1 or higher. We selected the local response at four days for our study as this time point gave the most significant overlap with WikiPathways.

We retrieved all human and mouse signaling pathways from the WikiPathways database [17, 18]. 25 pathways with at least 4 DE genes in both human and mouse were selected (see a table in Additional file 1).

Three pathways from this table were visualized using SyNDI to illustrate how its synchronous network visualization functionality provides an easy and effective approach to compare pathways between human and mouse. Detailed step by step instructions to run these examples are provided in Additional file 2. All needed scripts and data files are provided in Additional file 3.

### Complement and coagulation cascades

**Fig. 4:**
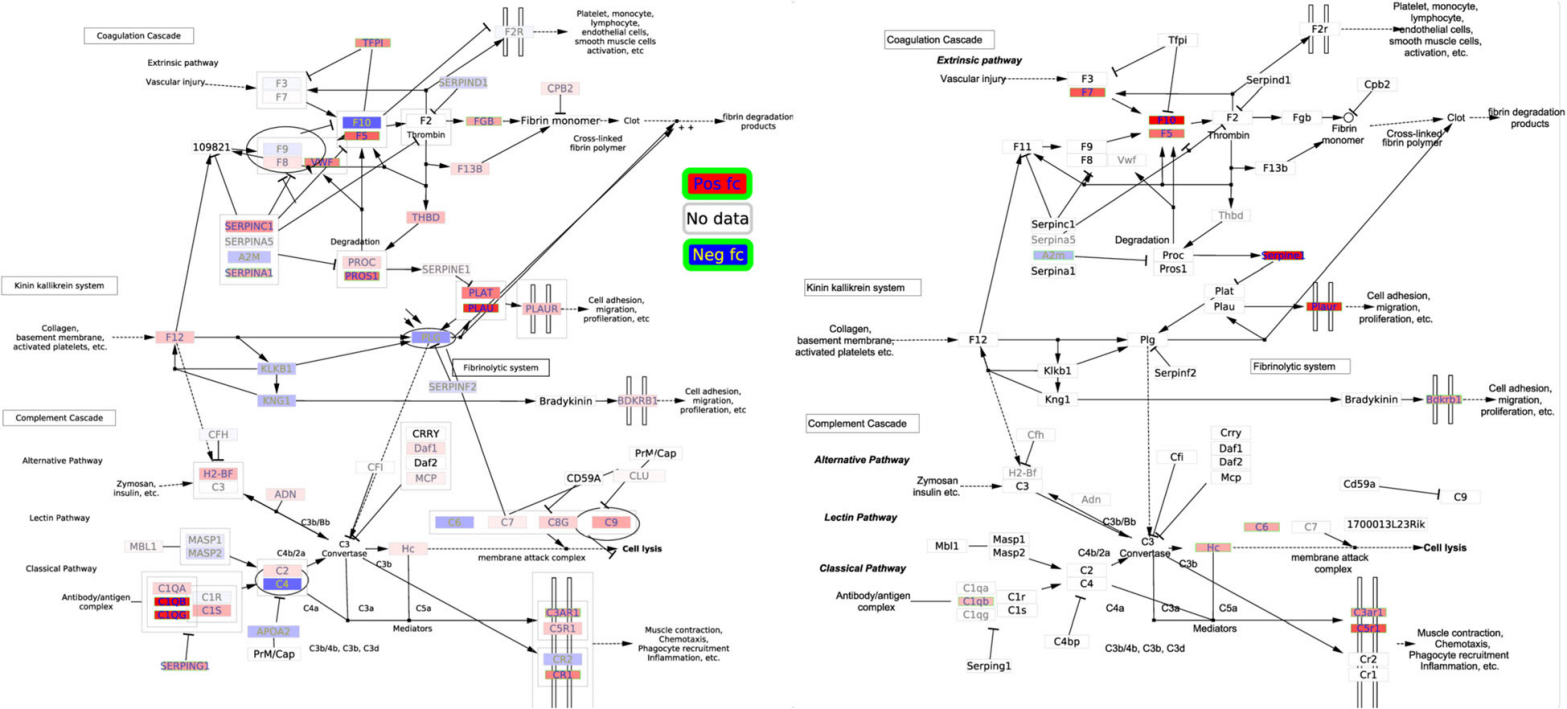
DE genes on “Complement and Coagulation Cascades” pathway upon *S. aureus* infection, human pathway on the left part and mouse on the right. Node color has been mapped to log2 fold change; red/blue denoting positive and negative values respectively (see legend). White color is used for nodes (genes or metabolites) for which either no data was available or changes were not deemed significant. The human pathway contains 169 nodes and 100 edges and the mouse pathway 148 nodes and 86 edges. Additional_file_7.zip contains a Complement_and_Coagulation_Cascades_human_mouse.cys file which can be opened on Cytoscape to view these pathways with better resolution

As indicated in Additional file 1, this pathway (Fig. 4) has one of the largest number of DE genes among those already reported in the literature to be differentially regulated in both human and mouse blood samples under various injury or bacterial infection conditions (including *S. aureus* infection). Nearly all DE genes in this pathway were up-regulated. The complement system and coagulation system are main columns of innate immunity and hemostasis respectively [26], so their up-regulation in human and mouse indicated an attempt of the hosts to fight against injuries or infections and to recover from damage. Among those 12 DE genes in this pathway found in human and mouse datasets, only 3 genes (F5, C1QB, and C3AR1) are homologs and they appear significantly up-regulated in both cases. Using SyNDI’s synchronous visualization, one can immediately identify that C1QB and C3AR1 belong to the classical pathway of the complement cascade, but F5 is among several other up-regulated genes in the coagulation cascade. C1QB is a subcomponent subunit of C1Q. Deficiency of C1q has been reported to be associated with recurrent infections among Inuit people [27]. Literature studies about C3AR1 and bacterial infection are very limited. Antunes and Kassiotis [28] studied influenza A virus infection-induced pathology in lymphocyte-deficient mice. C3ar1 in cells of the monocyte/macrophage lineage was one of the most highly induced gene transcripts, suggesting a role of C3ar1 in infection. F5 is a central regulator of hemostasis. In mice, reduction of F5 in blood plasma or platelet caused higher mortality upon Group A *Streptococcus* infection, highlighting the importance of F5 pool in host defense [29]. Overall, this visualization feature has facilitated quick identification of common regulation trends in parts of the complement and coagulation cascades between human and mouse. It can also speed up comparison of DE genes which are different between the two species in this pathway for potential further investigation.

### Wnt signaling pathway and pluripotency

**Fig. 5:**
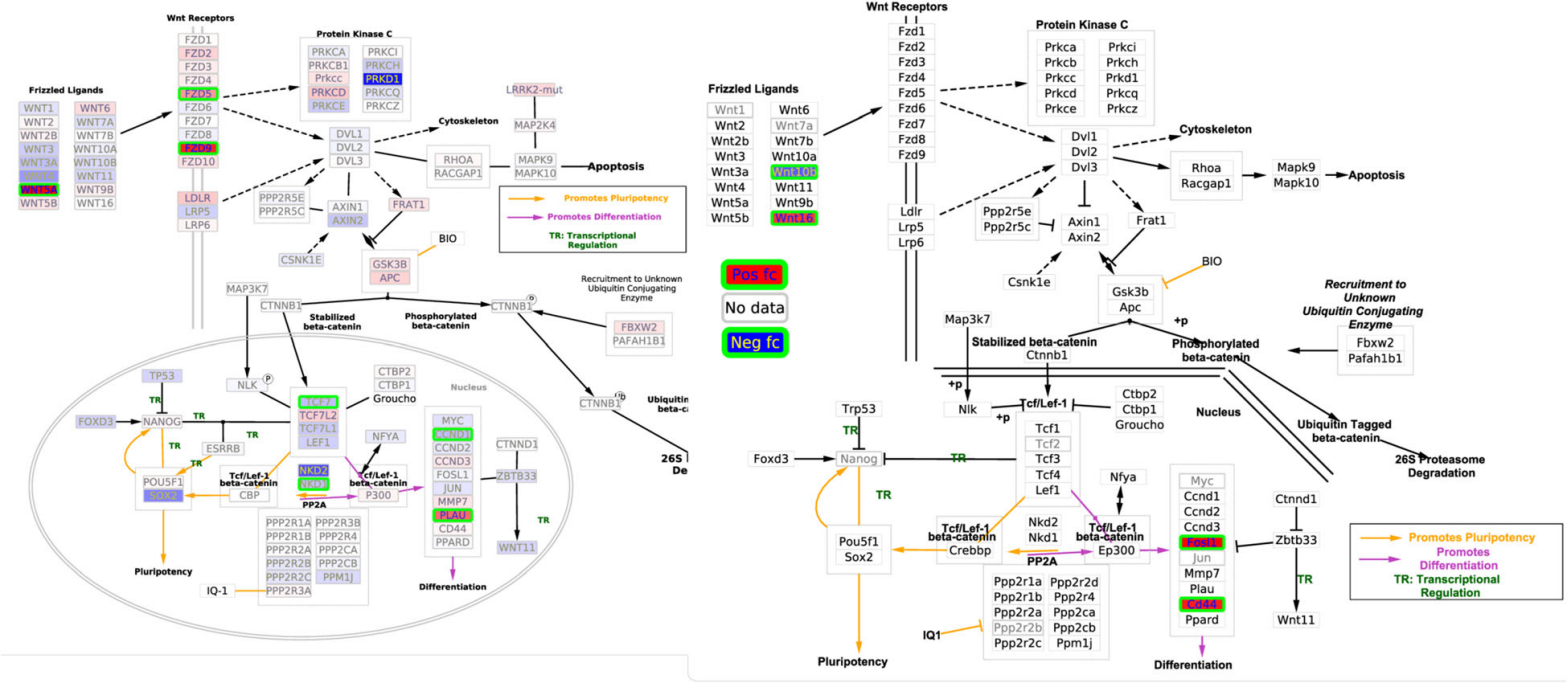
DE genes on “Wnt Signaling Pathway and Pluripotency” pathway upon *S. aureus* infection, human pathway on the left part and mouse on the right. See legend in Fig. 4 for additional information on coloring scheme. The human pathway contains 174 nodes and 55 edges and the mouse pathway 175 nodes and 54 edges. Additional_file_7.zip contains a Wnt_Signaling_Pathway_and_Pluripotency_human_mouse.cys file which can be opened on Cytoscape to view these pathways with better resolution

The Wnt signaling pathway has been reported in several studies as commonly regulated in human and mouse [30, 31]. Wnt signaling are responsible for cell differentiation, development, and tissue homeostasis etc. [32, 33]. A direct evidence for the relevance of Wnt5A in severe systemic inflammation is supported by the finding of higher Wnt5A levels in patients with sepsis than in healthy individuals [32]. Although all those DE genes in this pathway are different in human and mouse, from Fig. 5 we can easily identify that a few genes belonging to frizzled ligands and some of the beta-catenin target genes in the nucleus are differentially expressed in both mouse and human. It is expectable that differences between species would result in different genes being regulated in similar pathways in human and mouse. Those commonly regulated sub-networks of the Wnt signaling pathway and pluripotency network as shown by the synchronous visualization are tentative leads for further investigation of common signaling mechanisms in human and mouse upon *S. aureus* infection.

### Insulin signaling

**Fig. 6:**
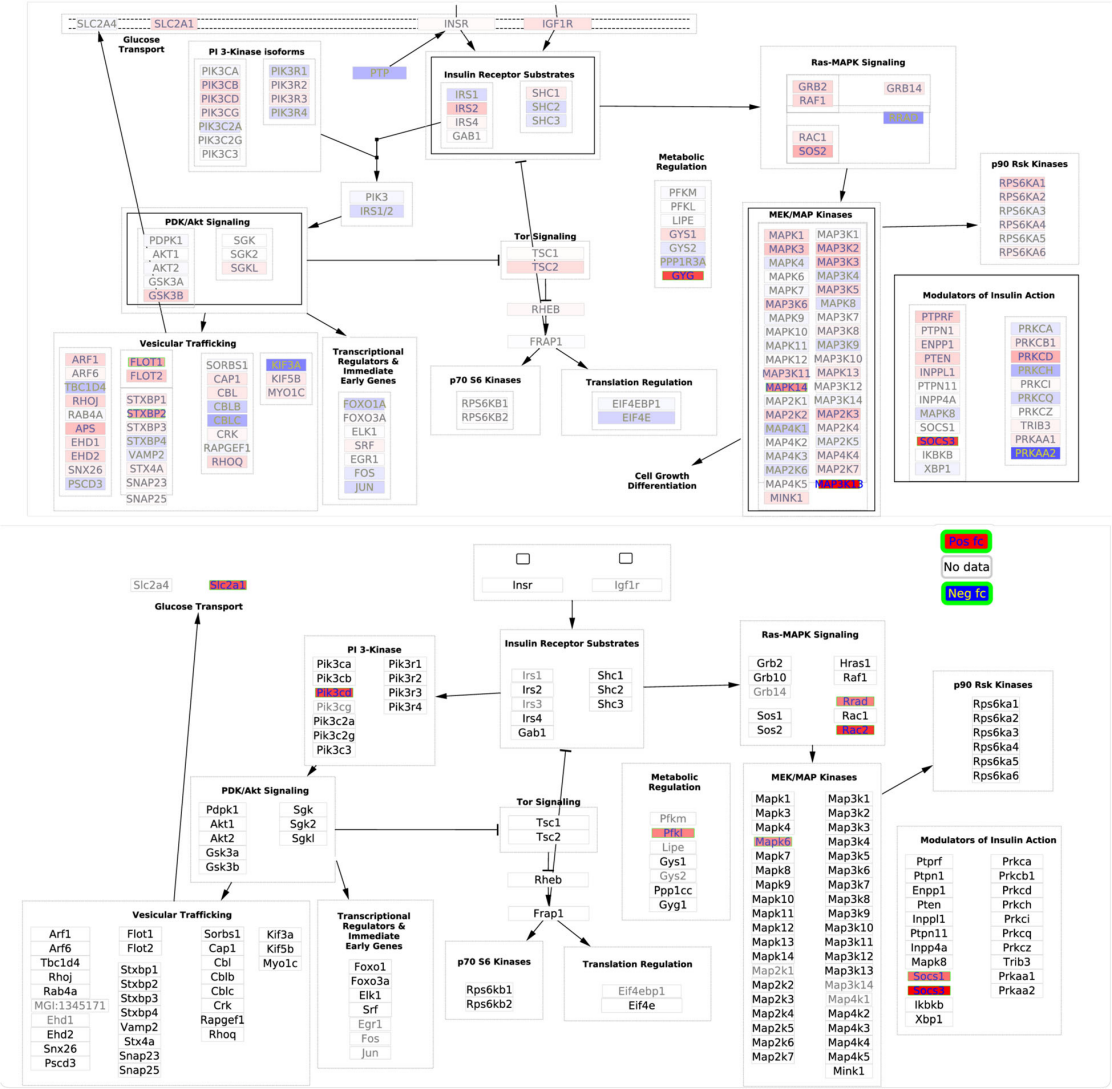
DE genes on “Insulin Signaling” pathway upon *S. aureus* infection, human pathway on the top part and mouse on the bottom. See legend in Fig. 4 for additional information on coloring scheme. The human pathway contains 226 nodes and 25 edges and the mouse pathway 195 nodes and 15 edges. Additional_file_7.zip contains an Insulin_Signaling_human_mouse.cys file which can be opened on Cytoscape to view these pathways with better resolution

The insulin signaling pathway contains 8 DE genes in human and in mouse, of which only SOCS3 is shared between the two species (Fig. 6). All these DE genes were up-regulated in both species. Georgel et al. reported TLR2 affected the outcome of mouse skin infection by bacteria [34]. In a study of gut microbiota of type 2 diabetes and obesity subjects, it was observed that TLR2 and inflammatory pathways were activated in obese individuals and insulin signaling was impaired relative to lean individuals [35]. Although the involvement of insulin signaling in diabetes is well-known, the potential role of this pathway in bacterial infections is rarely studied in the literature. Mele and Madrenas [36] studied literature evidence of infections by *S. aureus* and suggested TLR2 signals can differentially induce SOCS1 and SOCS3. In Fig. 6, both Socs1 and Socs3, belonging to modulators of insulin action, were significantly up-regulated in mouse. Further investigation is necessary to verify the potential relationship between *S. aureus* infection and insulin signaling pathway, but the network visualization approach has provided a convenient method to identify pathway candidates that appear to share unknown connections.

### Identification of binding motifs associated to DosR in *M. tuberculosis*

A pipeline for the reconstruction of gene co-expression networks from a compendium of expression data was described in [16] to where we refer the reader for additional details. This pipeline is highly customizable and its default values correspond to the following brief description. From a gene expression compendium, similarity between gene expression profiles is scored using Pearson’s correlation for each gene pair. The significance of the similarity is scored using an estimate for the null model based on the rest of the similarity scores obtained for the members of the pair evaluated independently [37]. A generalization of the data processing inequality is iteratively applied to prune possible spurious associations from the network [38]. Stand-alone scripts implementing this pipeline can be retrieved from Additional file 3 of [16].

**Fig. 7:**
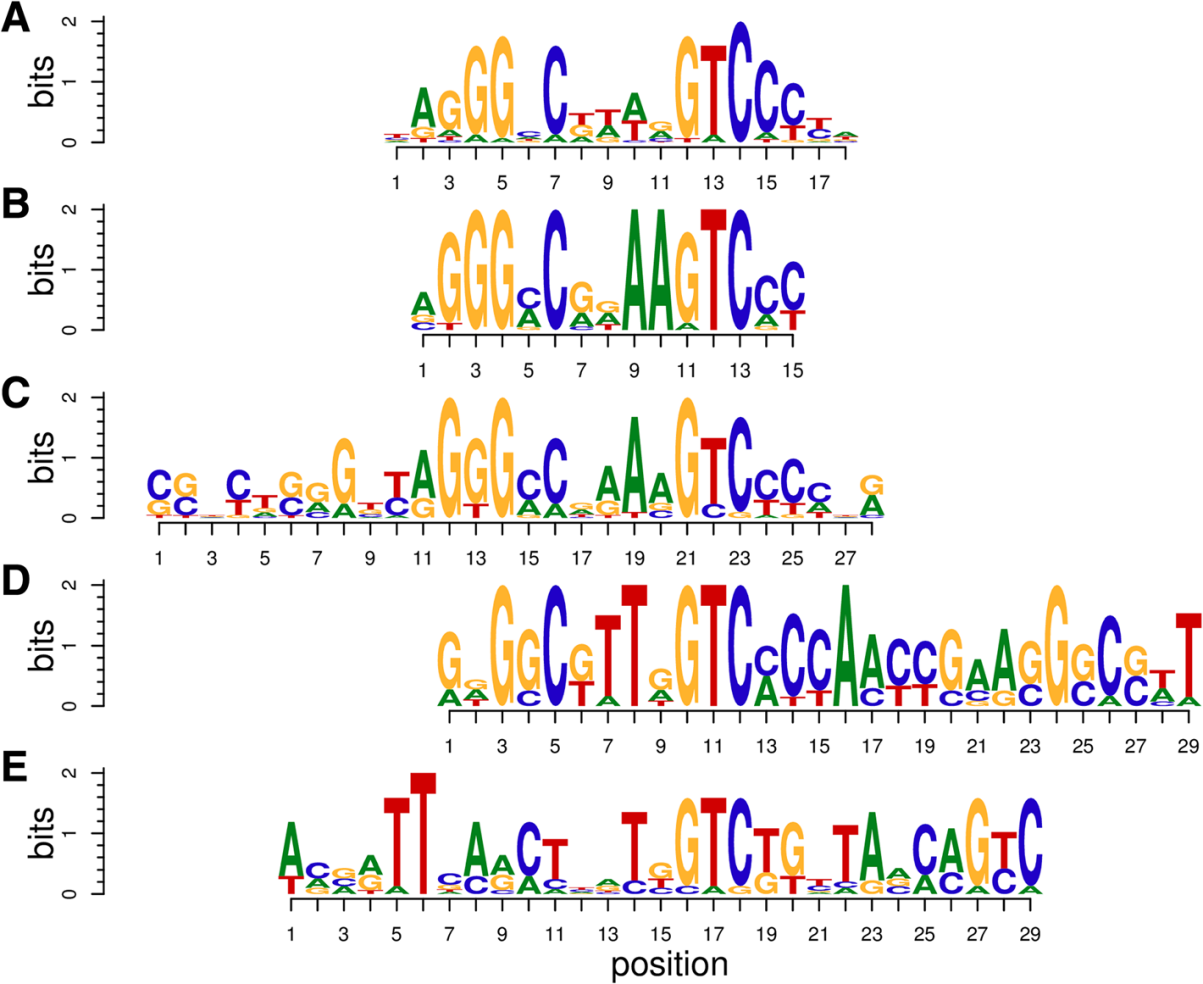
Comparison of DosR and ESX-1 related motifs**. a** DosR motif as reported in [48] (**b**) Exploration path 3 motif. **c** Exploration path 2 motif 2. **d** Exploration path 1 motif. **e** Exploration path 2 motif 1

We have used the Meme2Fimo tool to investigate transcriptional regulation of *M. tuberculosis*, the aetiological agent of tuberculosis. Specifically we investigated the role and regulation of ESX-1 associated genes *espA*, *C* and *D* and the role of DosR in regulating these genes. ESX-1 is a type VII secretion system required for the secretion of virulence proteins such as EsxA (ESAT-6) and EsxB (CFP-10). These are involved in immune modulation and phagosome escape [39–41]. EspACD is required for EsxA-EsxB secretion and pore formation [42, 43]. Multiple regulators such as PhoP, EspR, MprA, CRP are involved in modulation of ESX-1 and its secreted factors [44]. The transcription factor DosR (DevR) mediates the hypoxic response of *M. tuberculosis* and triggers the onset of dormancy which enables long term survival of the bacteria within the lung granulomas of the human host [45]. DosR regulon is essential for persistence and pathogenesis of *M. tuberculosis* [46]. ChipSeq experiments initially identified over 600 gene targets for DosR [47] and its binding motif is shown on Fig. 7 [48]. Integration of heterogeneous molecular networks with this data led to the identification of five groups of genes with distinct expression profiles among this initial set [16].

Here we used SyNDI framework to further investigate additional regulatory motifs related to ESX-1 systems by simultaneous exploration of the *CLR*, S*TRING.db fusion, STRING.db neighbourhood*, *operon* and *BLAST based homology (bbh)* networks presented in [16], to where we refer the reader for additional information on these networks. Technical details are provided in Additional files 4 and 5.

### Exploration path 1: ESX-1 associated genes *espA,C* and *D*

Initially, ESX-1 related genes, *espACD*, and other closely positioned genes in the CLR network were selected. The gene selection was transferred to the fusion network and three additional genes were identified in their neighbourhood. This selection was further enlarged with genes in their neighbourhood previously reported in the DosR regulon [16]. Transferring the selection to the *bbh* network led to the identification of three pairs of homologous genes. In each pair one gene belongs to the ESX-1 related gene set whereas the other one is in the DosR regulon (see Table 1). In the fusion network genes in these homology pairs within the DosR regulon appear as a densely connected cluster, together with Rv0080 and TB31.7. TB31.7 is a universal stress protein family protein responding to stress signals and has been shown to be involved in growth arrest during latent infection.

**Table 1:** Hypothetical homologous complexes

ESX-1 cluster related	DosR cluster related
*Rv0569*	*Rv2302*
*Rv2632c*	*Rv1738*
Rv2406c*	Rv2626c*
	*Rv0080*
	*TB31.7*

Pairs of homolog genes in the ESX-1 and DosR related clusters of two hypothetical homologous complexes. * low similarity (E-value 3e-09 < network visualization threshold)

To further investigate the role of TB31.7 a new selection was made in the *bbh* network by adding six TB31.7 homologs, five of which are in the DosR regulon. Meme2Fimo was iteratively used to explore upstream sequences of these genes. Finally, a conserved motif similar to the one reported for DosR was identified (Fig. 7). However, some distinct features appear showing that regulation of ESX-1 related genes *espACD* is complex, integrating signals from hypoxia via DosR but also possibly increased cell stress signals via TB31.7 homologs.

### Exploration path 2: TB31.7 and its homologs

To further investigate the *TB31.7* gene and its homologs, we selected them and neighbouring genes within the *neighbourhood* network. Upstream regulatory regions analysis lead to the description of another motif (Fig. 7). A subset of genes (*Rv2621c, Rv2622*), coding for a possible transcriptional regulator and methyltransferase, with this motif in their upstream regions appear in the CLR network with a cluster of genes related to mycolic acid synthesis. The ratio of free and bound mycolic acids is known to change under hypoxia and cell wall stress [47].

We further investigated the DosR regulation of Universal Stress Protein (USP) homologs to TB31.7 and its relation to ESX-1. We described another motif in Fig. 7.

### Exploration path 3, likely sigE binding motif

We explored the DosR regulon to identify elements with additional regulatory influences. USPs homologs to TB31.7 with the DosR regulon and genes in the same operons were selected. Transferring the selection to the gene *neighborhood* network showed the relationship between these two related groups and suggested some genes to be further included in the selection. Yet another motif (Fig. 7) was described in the upstream regions of these genes.

This motif is similar to the binding motif of the AlgU sigma factor from *P. aeruginosa* which is homologous to SigE in *M. tuberculosis* [49]. SigE and SigH together with MprAB function to detect and protect against cell stress such as misfolded proteins, heat shock, acidic pH, exposure to detergent, and oxidative stress. These conditions are associated with failed immune modulation which is related to the DosR regulated dormancy regulon [50–52]. Moreover, Rv0080, which is also in the DosR regulon, has been reported as a regulatory hub of the hypoxia response regulated by MprA [47, 53].The identified binding motif shows similarity to the motifs detected upstream of genes experimentally shown to be regulated by SigE and SigH regulated genes [54].

### Motif comparison

**Fig. 8:**
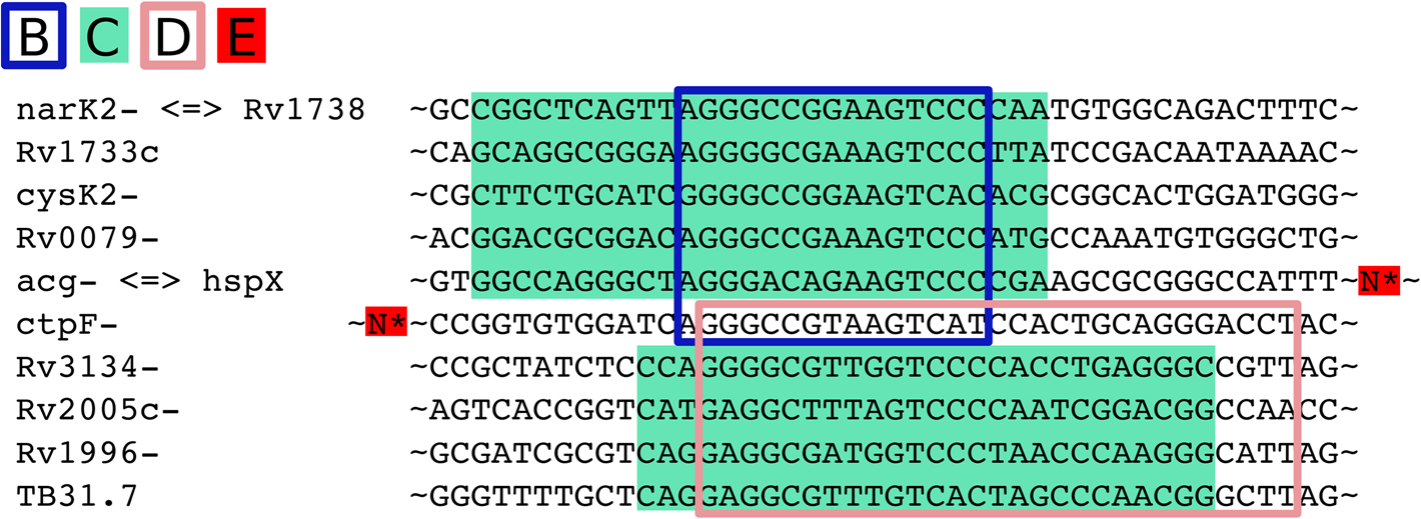
Shifted motif alignment**.** Marked region denotes the region containing the sequence to which the motif matches. The regions marked for the motif D regions are shifted. See Fig. 7 for the legend

**Fig. 9:**
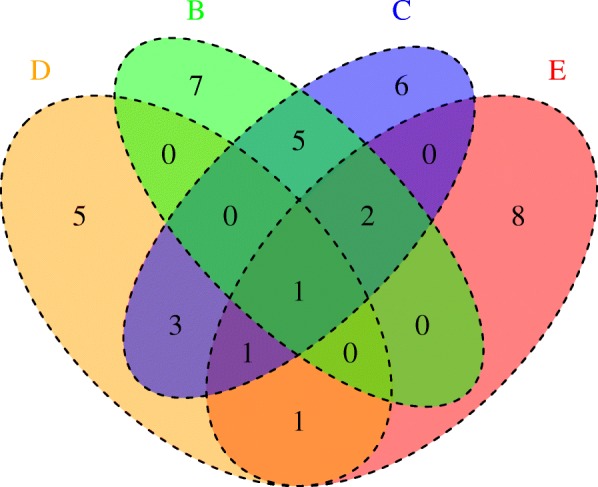
Shared genes. Presence of binding motifs A, B, C and D in gene upstream regions. See Fig. 7 for Legends A, B, C and D motif description

Figure 7 shows five related binding motifs. The location of these motifs is shown in Fig. 8 and Additional file 6. The groups of genes controlled by this motifs are shared as shown in Fig. 9 Fig. ee. Inspection of the locations of the motifs shows their overlaps in the upstream regions of the various shared genes of motifs B, C and D, which indicates that the shifted motifs might still be functional. The general DosR motif GGGNCNNNNGNCCC is palindromic, whereas motif B GGGNCNN**AA**G**T**C has a unique element, which is not palindromic. Both SigE and DosR are related to the modulation of process directly related to growth within human macrophages, the similarity between this motif and the AlgU motif in *P. aeruginosa* led us to hypothesize that DosR and SigE can bind to the same regions. Furthermore motif D GGGNCN**TT**NG**T**C also has a unique element, NAA in motif B is replaced by TTN.

The palindromic motif E lacks the characteristic GGGNCNNNNGNCCC pattern describing the general DosR binding motif. Only the GTC is conserved in comparison to the other motifs. The regions it matches are close (14 and 37 nucleotides) to the regions matched by motif B. Therefore we hypothesize that this motif might be associated to additional regulatory elements.

### Scalability of network visualization

SyncVis scales quite well for visualizing synchronously large networks (i.e. networks with a few thousands nodes and edges). In other, words it is possible to upload multiple networks of these sizes to Cytoscape and then select a specific nodes. SyncVis can then successfully highlight these nodes on all networks.

**Fig. 10:**
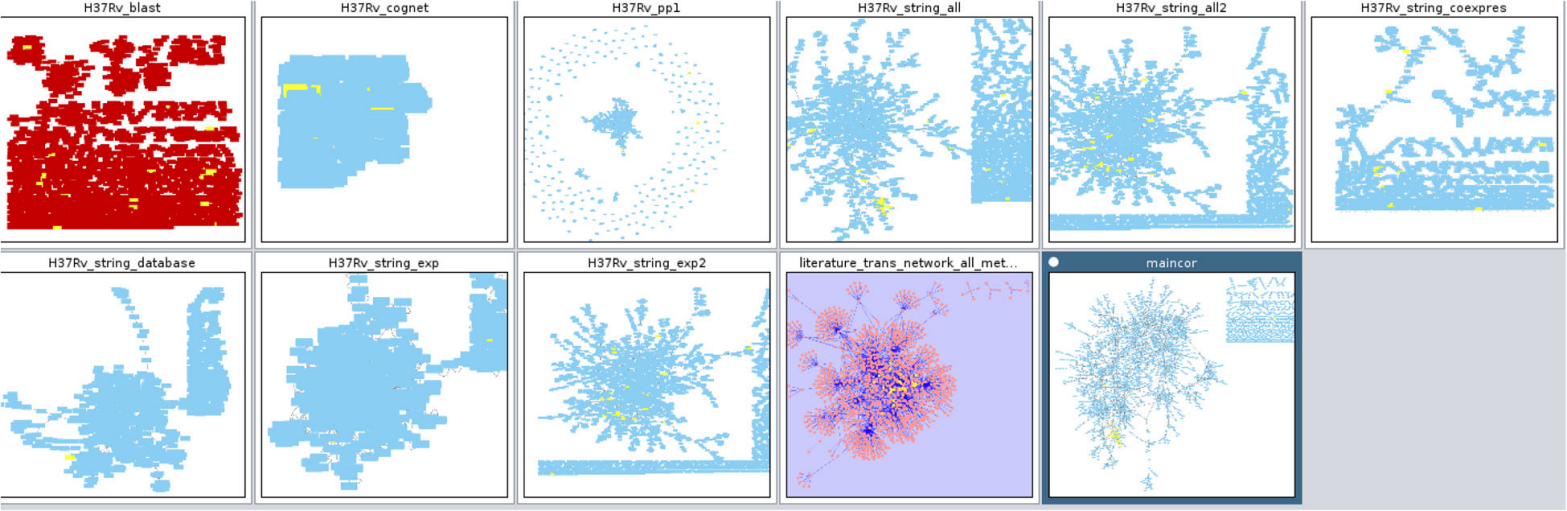
Scalability of network visualization. This figure illustrates a synchronous visualization of 11 big networks. The selected nodes are highlighted by yellow in all networks. The exact sizes of the networks are displayed in Table 2

In order to demonstrate this scalability, we have constructed a synchronous set of 11 networks on a ordinary desktop computer and then upload 11 gene identifiers from a file that were automatically in all networks. This visualization is presented in Fig. 10 and the network sizes are presented in Table 2. This construction is presented in detail in Additional file 7.

**Table 2:** The sizes of networks in Fig. 10

Network name	# nodes	# edges
H37Rv_blast	1705	2855
H37Rv_cognet	894	51718
H37Rv_pp1	1681	7446
H37Rv_string_all2	2919	7222
H37Rv_string_all	3020	7562
H37Rv_string_coexpres	1159	1904
H37Rv_string_database	853	4216
H37Rv_string_exp	451	1415
H37Rv_string_exp2	3020	7562
literature_trans_network_all_methods	1304	2132
maincor	2616	6072

The first column contains the network names, the second columns contains the number of nodes in the network, and the third column contains the number of edges in the network

However it is good to keep in mind these networks tend to be clumsy, so it is not easy to browser them. If the user wants to gain detailed biological insight from them, then perhaps she should restrict to specific sub-networks such as specific signaling pathways presented in the “Synchronous visualization of differentially expressed genes under *S. aureus* infection on human and mouse signaling pathways” section.

### Comparison with other tools, limitations and future directions

SyncVis is an integral part of SyNDI. SyncVis uses Cytoscape core for visualizing multiple networks. All generic development work done in the Cytoscape community will thus automatically be manifested in SyncVis. Moreover, the user of SyncVis can easily use other Cytoscape apps; for example there are some apps for advanced network visualization such as yFiles Layout Algorithms (https://apps.cytoscape.org/apps/yfileslayoutalgorithms), network comparisons [55, 56] and most importantly for network based biological analysis such as the ones illustrated in [57]. Tools like NAViGaTOR [58], Pajek [59] or igraph [60] are ideal for visualizing and/or analysing large networks but we have decided to implement SyncVis as a Cytoscape app due to the huge community effort behind Cytoscape and the continuous community support to biology oriented applications.

For the time being, SyncVis contains an automatic connection to only a few selected tools on the Galaxy platform. Some of the tools deployed in the presented use cases require collection of information that specifically relates to the studied organism, such as GO gene annotation and upstream sequence information for each gene. This information is derived from the genome but requires additional bioinformatics analysis or database mining for each organism and different tools have to be used for fungi, bacteria, mammals and so forth. We have chosen not to include the retrieval of this information as part of the SyNDI framework, which might limit its application.

A potential future direction is to connect SyncVis to tools for genome analysis such as GenomeSpace [61] or SAPP [62]. The modular design of SyNDI allows addition of more of these tools.

Finally, in the current version, the user has to install additional software components to use SyNDI’s workflow. This could be streamlined by providing a script that installs all of these components.

## Conclusions

Here we have presented SyNDI, which is a framework that connect a user-friendly Cytoscape application for synchronous network representation to advanced additional analysis tools for example through a Galaxy interface.

We have showed the potential of such a framework through three use cases. Firstly we have shown how the synchronous SyNDI framework facilitates differential network analysis and how dedicated layouts can help pinpoint altered metabolites’ connectivity patterns at different levels of cardiovascular disease risk. Specifically such representations clearly emphasizes the altered interplay between amino acids and glucose at high latent risk.

Secondly, we have used SyNDI to compare common inflammatory response pathways in human and mouse by synchronous visualization of differentially expressed genes. We have visualized *S. aureus* infection transcriptomics data from human and mouse on signaling pathways. Most interestingly, inspection of the insulin signaling pathway a potential role of TLR2, which can induce SOCS3, in induction of inflammatory pathways in *S. aureus* infection even though there are so far very limited amount of studies to explain why insulin signaling is regulated in bacterial infection.

Finally, we have shown how SyNDI can be used to explore and better understand complex regulated systems such as ESX-1 and associated virulence proteins in *M. tuberculosis*. In addition we were able to detect multiple and related binding motifs within the DosR regulon which have not yet been described in the literature, including a motif that we hypothesize it is related to *M. tuberculosis* SigE.

Galaxy enables further development of SyNDI, so that additional analysis modules can be added and complemented with network visualization. Here only omics data has been used, but other data types (such as text mining results) and dedicated analysis tools can be seamlessly integrated within the framework. Users can also easily customize SyNDI for their needs as they can incorporate additional datasets to Galaxy and networks for visualization.

SyNDI provides a framework to visually inspecting local connections from multiple networks, regardless of their origin. Additionally, SyNDI integrates network visualization and and analysis through Galaxy. This represents major advantages with respect to the use of the separate tools in isolations. First of all there is an increase in usability, as the user can easily run analysis by selecting nodes on networks without complicated file handling (e.g. copy-pasting rows and columns from an Excel sheet to another). The second major advantage is that SyNDI and most important, the Galaxy interface, allows the development of analysis workflows so that in-silico analysis can be stored and re-used upon addition of new datasets.

## Availability and requirements

The source code can be found at https://gitlab.com/elindfors/syndi and a link to the online user manual can be found at Cytoscape App Store http://apps.cytoscape.org/apps/syncvis. The source code for generating the biological examples can be found in Additional files 3 and Additional 5.

## Additional files

Additional file 1: List of pathways in WikiPathways with at least 4 DE genes in both human and mouse upons *S. aureus* infection. (*) Gene expression showed regulation among human and mouse burns, trauma, bacterial infections (sepsis), and mouse *Candida* infection [30]. (**) Common pathway in murine (sepsis mice infected by *S. aureus*) and human (patients diagnosed with sepsis) responses to infection [31]. The pathways highlighted in yellow were selected for visualization. (XLS 8 kb)

Additional file 2: Synchronous visualization of differentially expressed genes under *S. aureus* infection on human and mouse signaling pathways. In this additional file we present a step by step description of the analysis performed to explore signaling pathways involved in response to *S. aureus* infection on human and mouse signaling pathways. (PDF 53 kb)

Additional file 3: Source code and data files of the example presented in Additional file 2. This additional file is a zip package that contains all source code data files used in Additional file 2. Detailed descriptions of each of these files are presented in a README file included in this zip package (ZIP 16.2 MB)

Additional file 4: Exploration paths to investigate additional regulatory motifs related to ESX-1 systems. This file contains the exploration paths and instructions to run the scripts to obtain these paths. (PDF 59 kb)

Additional file 5 Files of the example presented in Additional file 4. This additional file is a zip package that contains all files used in Additional file 4. Detailed descriptions of each of these files are presented in a README file included in this zip package. (GZ 30411 kb)

Additional file 6: Overlap in genes regulated by five DosR and ESX-1 related motifs. Shared genes regulated by overlapping regulatory motifs B, C, D, E. (TSV 590 bytes)

Additional file 7: This additional file is a zip package that contains all files and folder that used to generate Fig. 10. Detailed descriptions of each of these files and folders are presented in a README file included in this zip package. (ZIP 16.9 MB)

## Abbreviations

API: Application Programming Interface
BiNGO: Biological Networks Gene Ontology tool
C1QB: Complement Chromosome 1q B chain
C3ar1: Complement component 3a receptor 1
CVD: Cardiovascular diseases
DE: Differentially Expressed
F5: Coagulation factor V
FDR: False Discovery Rate
GO: Gene Ontology
HDL: High-Density Lipoprotein
HTML: HyperText Markup Language
LDL: Low-Density Lipoprotein
NMR: Nuclear Magnetic Resonance
PCLRC: Probabilistic Context Likelihood of Relatedness based on Correlation
RNA-seq: Ribonucleic acid-sequencing
SBML: Systems Biology Markup Language
SIF: Simple Interaction Format
SOCS1: Suppressor of Cytokine signaling 1
SOCS3: Suppressor of Cytokine Signaling 3
SSTI: Skin and Soft Tissue Infection
SyncVis: Synchronous Visualizer
SyNDI: Synchronous Network Data Integration framework
TLR2: Toll-Like Receptor 2
USP: Universal Stress Protein
VLDL: Very-low-density lipoprotein
XGMML: eXtensible Graph Markup and Modeling Language
XML: Extensible Markup Language
