# Supplementary material for: SyNDI: synchronous network data integration framework
Source: BMC Bioinformatics. 2018 Nov 6;19:403. doi: 10.1186/s12859-018-2426-5 (PMC6219086; doi:10.1186/s12859-018-2426-5)
Supplement: Supplementary file 2 — Synchronous visualization of differentially expressed genes under S. aureus infection on human and mouse signaling pathways. In this additional file we present a step by step description of the analysis performed to explore signaling pathways involved in response to S. aureus infection on human and mouse signaling pathways. (PDF 53 kb) [file 12859_2018_2426_MOESM2_ESM.pdf]

Step by step description - Synchronous visualization of differentially expressed genes under *S. aureus* infection on human and mouse signaling pathways

We have tested these instructions in Ubuntu 16.04 LTS and we used R version 3.3.1 to run the R scripts.

1. SyNDI installation

In order to install SyNDI, we went through the following steps:

- We went to the installation folder of Cytospape
  We opened a Cystocape by giving the following command:
  - ./cytoscape.sh
- We selected “Apps -> App Manager… -> Install from File...”
- Then we got a file chooser in which we selected multinetvisapp-1.0.0-SNAPSHOT.jar

Them all user interface elements needed in SyNDI automatically appeared in a “SyNDI - Vis” and “SyNDI - Gal” tabs under Cytoscape's control panel.

In order to get familiar with SyNDI's synchronous visualization feature, we can first open one of the above mentioned cys file on SyNDI. Then the corresponding human and mouse networks are loaded. We can select all DE genes (the genes with green border) holding the control button down in a human network. Then we can go to the mouse network and see the corresponding mouse homologs are highlighted. The nodes with green border from these highlighted nodes are commonly DE genes. These nodes are C1qb, C3ar1 and F5 in case of “Complement and Coagulation Cascades”. You can do this procedure of course in the way around - first select DE genes in the mouse network and then go to the human network to see the corresponding mouse homologs.

2. Download transcriptomics data

2.1 Download human transcriptomics data from GEO

We downloaded GPL6947-13512.txt from
http://www.ncbi.nlm.nih.gov/geo/query/acc.cgi?acc=GPL6947
-> "Download full table..."

We downloaded GSE30119_series_matrix.txt.gz from
http://www.ncbi.nlm.nih.gov/geo/query/acc.cgi?acc=GSE30119
-> Download family -> Series Matrix File(s)
and extracted it.

We will henceforth denote the folder in which the above mentioned files were stored by $HUMAN_GE_FOLDER

2.2 Download mouse transcriptomics data

We downloaded pone.0124877.s001_Table_S1.xls from
http://www.ncbi.nlm.nih.gov/pmc/articles/PMC4406450/
-> "Supporting Information" -> "S1 Table"

Direct link:
http://www.ncbi.nlm.nih.gov/pmc/articles/PMC4406450/bin/pone.0124877.s001.xls

We created a text file "Table_S1_Day_4_LOCAL.txt" and copied the content of "Day 4 LOCAL" sheet from the downloaded excel file into this text file.

We will henceforth denote the folder in which the above mentioned files are stored by $MOUSE_GE_FOLDER

3. Download WikiPathways data

We downloaded the following zip packages from http://www.wikipathways.org/index.php/Download_Pathways on December 1, 2015:

- wikipathways_Homo_sapiens_Curation-AnalysisCollection__gpml.zip
- wikipathways_Mus_musculus_Curation-AnalysisCollection__gpml.zip

We extracted the above mentioned zip packages with the following commands:

- unzip wikipathways_Homo_sapiens_Curation-AnalysisCollection__gpml.zip
- unzip wikipathways_Mus_musculus_Curation-AnalysisCollection__gpml.zip

We henceforth denote the folders in which the above mentioned files were extracted by $HUMAN_WP_FOLDER and $MOUSE_WP_FOLDER, respectively.

4. Modify the WikiPathways files

The WikiPathways files are in GPML format that are not easy to handle in R environment. We therefore impelemented a stand-alone java application called “AllWikiPathwaysGeneIdsRetrieval”. This application retrieves all gene ids for all species specfic WikiPathways and puts them in a table format in which rows contain pathways and columns contain identifiers of genes involved in the pathway and other pathway specific meta data.
We used the following commands to run this application:

- java -cp AllWikiPathwaysGeneIdsRetrieval.jar de.lg.allwikipathwaysgeneidsretrieval.AllWikiPathwaysGeneIdsRetrieval $HUMAN_WP_FOLDER $HUMAN_WP_FOLDER/Human_wikipathways.txt
- java -cp AllWikiPathwaysGeneIdsRetrieval.jar de.lg.allwikipathwaysgeneidsretrieval.AllWikiPathwaysGeneIdsRetrieval $MOUSE_WP_FOLDER $MOUSE_WP_FOLDER/Mouse_wikipathways.txt

In order to convert entrez gene ids to ensembl ids and vice versa, we implemented another stand-alone java applicaton called “ WikiPathwaysGeneIdConverter”.
We used the following commands to run this application:

- java -cp WikiPathwaysGeneIdConverter.jar de.lg.wikipathwaysgeneidconverter.WikiPathwaysGeneIdConverter $HUMAN_WP_FOLDER/Human_wikipathways.txt $HUMAN_WP_FOLDER/Human_wikipathways_ids.txt true
- java -cp WikiPathwaysGeneIdConverter.jar de.lg.wikipathwaysgeneidconverter.WikiPathwaysGeneIdConverter $MOUSE_WP_FOLDER/Mouse_wikipathways.txt $MOUSE_WP_FOLDER/Mouse_wikipathways_ids.txt false

If you want to look into the source code of the above mentioned java applications, you can use the following commands to extract the jar packages:

- jar xf AllWikiPathwaysGeneIdsRetrieval.jar

- jar xf WikiPathwaysGeneIdConverter.jar

5. Prepare node attributes for human and mouse

We used CreateHumanNoas.R and CreateMouseNoas.R to generate node attributes for the visualization of signaling pathways by giving the following commands on the R command prompt:

- Rscript --vanilla CreateHumanNoas.R $HUMAN_GE_FOLDER/GPL6947-13512.txt $HUMAN_GE_FOLDER/GSE30119_series_matrix.txt
- Rscript --vanilla CreateMouseNoas.R $MOUSE_GE_FOLDER/Table_S1_Day_4_LOCAL.txt

The above mentioned scripts outputted HumanNoas.txt and and MouseNoas.txt. We stored them in folders that we henceforth denote by $HUMAN_ATTR_FOLDER and $MOUSE_ATTR_FOLDER, respectively.

6. Construction of Additional file 1

First we needed to map the WikiPathways between human and mouse.
We run the following R script from the command line:

- Rscript --vanilla FindEquivWps.R $HUMAN_WP_FOLDER/Human_wikipathways_ids.txt $MOUSE_WP_FOLDER/Mouse_wikipathways_ids.txt

The above mentioned script compared pathways based on exact match (i.e. considered pathways to be same only if the have exactly same name). It ouputs Equiv_wps.txt, Non_equiv_human_wps.txt and Non_equiv_mouse_wps.txt. In order to get more extensive mapping, we browsed the content of Non_equiv_human_wps.txt and Non_equiv_mouse_wps.txt manually and identified a few other mappings between human and mouse WikiPathways. A complete list of these mappings is at Manual_matches_wps.txt. We then created Equiv_wps_ext.txt by appending Manual_matches_wps.txt to Equiv_wps.txt. We henceforth denote the folder in which these file are by $EQUIV_WPS_FOLDER.

Then we run the following R script from the command line in order to produce the content of Additional file 1 in the main text:

- Rscript --vanilla FindWpsWithAtLeast4DeGenes.R $WP_FOLDER/Human_wikipathways_ids.txt $EQUIV_WPS_FOLDER/ $WP_FOLDER/Mouse_wikipathways_ids.txt $HUMAN_ATTR_FOLDER/HumanNoas.txt $MOUSE_ATTR_FOLDER/MouseNoas.txt $EQUIV_WPS_FOLDER/ Equiv_wps_ext.txt

The results are stored Human_mouse_at_least_4_de_genes.txt from which we copy-pasted columns to Additional file 1.

7. Visualize pathways on SyNDI

We selected the following WikiPathways for visualization:

- Complement and Coagulation Cascades
- Wnt Signaling Pathway and Pluripotency
- Insulin Signaling

In order to polish the visualization, we used a text editor and PathVisio (http://www.pathvisio.org/) to modify the GPML files of all above mentioned WikiPathways except the Insulin Signaling from mouse (since this was clean enough). The contents of the modified files are in the following GPML files:

- Hs_Complement_and_Coagulation_Cascades_WP558_79680_modif.gpml
- Mm_Complement_and_Coagulation_Cascades_WP449_71733_modif.gpml
- Hs_Wnt_Signaling_Pathway_and_Pluripotency_WP399_79474_modif.gpml
- Mm_Wnt_Signaling_Pathway_and_Pluripotency_WP723_80030_modif.gpml
- Hs_Insulin_Signaling_WP481_82731_modif.gpml

For the Insulin Signaling from mouse, we used its original format:

- $MOUSE_WP_FOLDER/Mm_Insulin_Signaling_WP65_82885.gpml

We used the following XML file in order to import styles for visualization:

- Human_Complement_and_Coagulation_Cascades_style.xml
- Mouse_Complement_and_Coagulation_Cascades_style.xml
- Human_Wnt_Signaling_Pathway_and_Pluripotency_style.xml
- Mouse_Wnt_Signaling_Pathway_and_Pluripotency_style.xml
- Human_Insulin_Signaling_style.xml
- Mouse_Insulin_Signaling_style.xml

We visualized these pathways on SyNDI by going through the below mentioned steps, separately for human and mouse version of WikiPathway in the same Cytoscape session.

- We loaded the GPML files into Cytoscape by using a short-cut "ctrl + L", or selecting "File -> Import -> Network -> File ..." menu. Then we got a file chooser in which we selected the GPML file (e.g. Hs_Complement_and_Coagulation_Cascades_WP558_79680_modif.gpml) we want to visualize.
- Then we got a pop-up window asking if we would like to import the WikiPathways as Pathway or Network. We selected Pathway since we found it biologically more descriptive.
- We imported node attributes by selecting “File - > Import - > Table - > File...” menu. Then we got a file chooser in which we selected $HUMAN_ATTR_FOLDER\HumanNoas.txt (for a human pathway) or $MOUSE_ATTR_FOLDER\MouseNoas.txt (for mouse). Then we got a “Import Columns From Table” pop-up window. In this window we selected “XrefId” in the “Key Column for Network:” combobox. We did this whole node importing procedure separately by selecting “Enterez.ID” and “Ensembl.ID” for key column.
- We imported a style for visualization by selecting the “File - > Import - > Style” menu. Then we got a file chooser in which we selected an XML file corresponding the visualized WikiPathway. The imported visualized style appeared then under the “Style” tab in Cytoscape. When we selected the imported style, the visualized WikiPathway got the legends explained in the main text figures.
- In the end we save the whole visualization in one Cytoscape session file. Since we handled three pathway in total we ended up with the following cys files:
    - Complement_and_Coagulation_Cascades_human_mouse.cys
    - Wnt_Signaling_Pathway_and_Pluripotency_human_mouse.cys
    - Insulin_Signaling_human_mouse.cys
