# Supplementary material for: SyNDI: synchronous network data integration framework
Source: BMC Bioinformatics. 2018 Nov 6;19:403. doi: 10.1186/s12859-018-2426-5 (PMC6219086; doi:10.1186/s12859-018-2426-5)
Supplement: Supplementary file 4 — Exploration paths to investigate additional regulatory motifs related to ESX-1 systems. This file contains the exploration paths and instructions to run the scripts to obtain these paths. (PDF 59 kb) [file 12859_2018_2426_MOESM4_ESM.pdf]

Detailed exploration paths

Exploration path 1: ESX-1 associated genes espACD

1.1) Select genes within CLR network ESX-1 related cluster:
Rv3615c;Rv3613c;Rv1639c;Rv1387;Rv3612c;Rv2406c;Rv1386;Rv1284;Rv3616c;Rv2632c;Rv2302;Rv3614c

1.2) Fusion network select genes in neighbourhoudneighborhood:
Rv3615c;Rv3613c;Rv1639c;Rv1387;Rv3612c;Rv2406c;Rv1386;Rv1284;Rv3616c;Rv2632c;Rv2302;Rv3614c;Rv0143c;Rv3709c;Rv1738;Rv1293;Rv1294;Rv0080;Rv0569;Rv3341;Rv2623;Rv2837c;Rv2626c;Rv0056;Rv3907c

* See homology pairs in blast network (Table 1 in the main text)

1.3) New selection, select all homologs in bbh network of TB31.7:
Rv3134c;Rv2624c;Rv2623;Rv1996;Rv2005c;Rv2026c;Rv2028c

1.4) Naive operon extend:
Rv3134c;Rv2624c;Rv2623;Rv1996;Rv2005c;Rv2026c;Rv2028c;Rv3133c;Rv3132c;Rv3135;Rv3136;Rv3137;Rv3138;Rv3139;Rv3140;Rv3141;Rv1997;Rv2004c;Rv2003c;Rv2006;Rv2025c;Rv2024c;Rv2023c;Rv2022c;Rv2021c;Rv2020c;Rv2027c

1.5) Apply Meme2Fimo

1.6) Take top hits from Meme2Fimo result:
Rv1731;Rv3134c;Rv1997;Rv1996;Rv3135;Rv2006;Rv2623;Rv1730c;Rv2005c;Rv2023c;Rv2024c

1.7) Apply Meme2Fimo: motif D

1.8) Take gene with upstream containing the motif from Meme2Fimo result:
gabD2;Rv1730c;PPE50;Rv3134c;otsB1;Rv2005c;Rv1996;TB31.7;ctpF;Rv2024c;Rv2023c

Exploration path 2a: Extraction of two stress protein associated motifs

2a.1) New selection, reselect all homolgos in bbh network of TB31.7:
Rv3134c;Rv2624c;Rv2623;Rv1996;Rv2005c;Rv2026c;Rv2028c

2a.2) Select related genes in neighbourhoodneighborhood network:
Rv0844c;Rv3134c;Rv3133c;Rv2624c;Rv2625c;Rv2626c;Rv3132c;Rv2620c;Rv2621c;Rv2627c;Rv0845;Rv1997;Rv1996;Rv1995;Rv2619c;Rv2032;Rv2006;Rv2004c;Rv2003c;Rv1992c;Rv1993c;Rv1994c;Rv2031c;Rv2030c;Rv2622;Rv2623;Rv2029c;Rv2005c;Rv2026c;Rv2025c;Rv2028c;Rv2027c

2a.3) Select extra related genes seen by subsequent numbering and also co-expression in CLR network:
Rv0844c;Rv3134c;Rv3133c;Rv2624c;Rv2625c;Rv2626c;Rv3132c;Rv2620c;Rv2621c;Rv2627c;Rv0845;Rv1997;Rv1996;Rv1995;Rv2619c;Rv2032;Rv2006;Rv2004c;Rv2003c;Rv1992c;Rv1993c;Rv1994c;Rv2031c;Rv2030c;Rv2622;Rv2623;Rv2029c;Rv2005c;Rv2026c;Rv2025c;Rv2028c;Rv2027c;Rv2617c

2a.4) Select extra related genes in neighbourhoodneighborhood network:
Rv0844c;Rv3134c;Rv3133c;Rv2624c;Rv2625c;Rv2626c;Rv3132c;Rv2620c;Rv2621c;Rv2627c;Rv0845;Rv1997;Rv1996;Rv1995;Rv2619c;Rv2032;Rv2006;Rv2004c;Rv2003c;Rv1992c;Rv1993c;Rv1994c;Rv2031c;Rv2030c;Rv2622;Rv2623;Rv2029c;Rv2005c;Rv2026c;Rv2025c;Rv2028c;Rv2027c;Rv2617c;Rv2618c

2a.5) Apply Meme2Fimo: motif E

2a.6) Take gene with upstream containing the motif from Meme2Fimo result:
Rv2618;Rv2617c;Rv1995;Rv1994c;otsB1;Rv2005c;acg;hspX;Rv0845;narL;Rv2622;Rv2621c;ctpF

Exploration path 2b, General DosR regulon, extraction of the likely sigE binding motif

2b.1) Select genes within CLR network ESX-1 related cluster:
Rv3615c;Rv3613c;Rv1639c;Rv1387;Rv3612c;Rv2406c;Rv1386;Rv1284;Rv3616c;Rv2632c;Rv2302;Rv3614c

2b.2) Deselect all known esx-1 associated genes:
Rv2406c;Rv2302;Rv2632c

2b.3) Select related genes fusion network, all 3 are in the same blob:
Rv2632c;Rv2302;Rv2406c;Rv0143c;Rv2623;Rv1738;Rv2837c;Rv2626c;Rv0056;Rv0080;Rv0569;Rv3341;Rv3709c;Rv1293;Rv1294;Rv3907c

2b.4) Select related genes in neighbourhoudneighborhood network, 3 genes are in one blob (TB31.7, RV1738,Rv0080):
Rv2632c;Rv2302;Rv2406c;Rv0143c;Rv2623;Rv1738;Rv2837c;Rv2626c;Rv0056;Rv0080;Rv0569;Rv3341;Rv3709c;Rv1293;Rv1294;Rv3907c;Rv2620c;Rv2621c;Rv007

8A;Rv2619c;Rv0079;Rv2748c;Rv2751;Rv2750;Rv2622;Rv1736c;Rv1737c;Rv1734c ;Rv1735c;Rv1732c;Rv1733c;Rv2749

2b.5) Select all homolgos in bbh network of TB31.7: Rv1738;Rv2632c;Rv2626c;Rv2620c;Rv2621c;Rv2302;Rv3341;Rv3709c;Rv2837c;Rv0078A;Rv2619c;Rv2406c;Rv0056;Rv0079;Rv0080;Rv3907c;Rv2748c;Rv2751;Rv2750;Rv1293;Rv1294;Rv0143c;Rv2622;Rv2623;Rv1736c;Rv1737c;Rv1734c;Rv1735c;Rv1732c;Rv1733c;Rv2749;Rv0569;Rv2026c;Rv3134c;Rv2624c;Rv1996;Rv2028c;Rv2005c

2b.6) Apply Meme2Fimo

2b.7) Take lost list of top hits from Meme2Fimo result: RV1733C;RV0079;RV1737C;RV1738;RV1996;RV2623;RV2005C; RV3134C;RV1735C;RV1734C;RV0569;RV2626C;RV1997;RV2825C;RV2031C;RV2032;RV3033;RV2338C;RV2339;RV0848;RV0961;RV0574C;RV1643;RV0667;RV1015C;RV2795C;RV1574;RV0522;RV1813C

2b.8) Q select(hold q key down while doing the selection) only genes in the Dos regulon related cluster in CLR network: Rv1738;Rv3134c;Rv2626c;Rv0574c;Rv1997;Rv1996;Rv0079;Rv1813c;Rv2032;Rv2031c;Rv2623;Rv2005c;Rv1737c;Rv1733c;Rv0569

2b.9) Apply Meme2Fimo

2b.10) Take top hits from Meme2Fimo result: RV1737C;RV1738;RV1813C;RV2031C;RV2032;RV0079;RV1996;RV0574C;RV2623;RV2005C;RV1733C;RV1997;RV3134C;RV3130C;RV3131;RV0848;RV0569;RV3409C;RV1628C;RV1629;RV2626C

2b.11) Apply Meme2Fimo: motif C

2b.12) Take gene with upstream containing the motif from Meme2Fimo result: Rv1738;narK2;acg;hspX;Rv3131;tgs1;Rv0079;TB31.7;Rv1813c;Rv2005c;Rv0574c;ctpF;Rv1996;cysK2;Rv3134c;Rv1733c;polA;Rv1628c

Exploration path 3, likely sigE binding motif

3.1) Continue from step 3.4: Rv2632c;Rv2302;Rv2406c;Rv0143c;Rv2623;Rv1738;Rv2837c;Rv2626c;Rv0056;Rv0080;Rv0569;Rv3341;Rv3709c;Rv1293;Rv1294;Rv3907c;Rv2620c;Rv2621c;Rv0078A;Rv2619c;Rv0079;Rv2748c;Rv2751;Rv2750;Rv2622;Rv1736c;Rv1737c;Rv1734c;Rv1735c;Rv1732c;Rv1733c;Rv2749

3.2) Q select only genes in the Dos regulon related cluster in CLR network:
Rv1738;Rv2626c;Rv0079;Rv0080;Rv2623;Rv1737c;Rv1733c;Rv0569;Rv2625c;Rv2624c;Rv0081;Rv0570

3.3) Naive operon extend:
Rv1738;Rv2626c;Rv0079;Rv0080;Rv2623;Rv1737c;Rv1733c;Rv0569;Rv2625c;Rv2624c;Rv0081;Rv0570;Rv1736c;Rv1735c;Rv1734c;Rv1732c;Rv0082;Rv0083;Rv0084;Rv0085;Rv0086;Rv0087;Rv0088;Rv0089;Rv0090;Rv0091;Rv0092

3.4) add select in neighbourhoodneighborhood connect 2 large groups & remove TB31.7 as neighbors are not in dos regulon:
Rv1738;Rv2624c;Rv2625c;Rv2626c;Rv2627c;Rv0079;Rv0081;Rv0080;Rv0083;Rv0082;Rv0086;Rv0087;Rv0084;Rv0085;Rv0088;Rv0089;Rv0090;Rv0092;Rv0091;Rv0570;Rv2628;Rv2629;Rv1736c;Rv1737c;Rv1734c;Rv1735c;Rv1732c;Rv1733c;Rv0569;Rv0567;Rv0568;Rv2631;Rv2630

3.5) Apply Meme2Fimo

3.6) Take top hits from Meme2Fimo result:
RV0079;RV1737C;RV1738;RV2031C;RV2032;RV1733C;RV2627C;RV2628;RV1735C;RV2629;RV1997;RV0089;RV0569;RV1734C

3.7 Apply Meme2Fimo: motif B

3.8) Take gene with upstream containing the motif from Meme2Fimo result:
Rv0079;Rv1738;narK2;acg;hspX;Rv2628;Rv2627c;ctpF;Rv1733c;Rv2629;cysK2;Rv1735c;Rv1734c;Rv0569;Rv0089

Instructions to run the exploration paths

Installation has been tested on ubuntu 16.04.

Setup Galaxy

Copy “setupGalaxy.sh” script and “SyNDITools” folder from Additional file 5 to a folder where you want to install Galaxy.
Installl Galaxy by running the following command:
./setupGalaxy.sh
This script creates a “galaxy” folder that contains everything you need to run this example on Galaxy.
Use run.sh to start the galaxy server. Server will be available at port 80.

Install SyNDI into Cytoscape

Open Cytoscape and select “Apps → App Manager...” menu. Then you will get a pop-up window, on which you should click on the “Install from File...” button. Then you will get a file chooser in which you should select multinetvisapp-1.0.0-SNAPSHOT.jar from Additional file 5.

Install Cytoscape session

Load the cytoscape session ‘MTB_H37Rv_set.cys’ file from the Additional file 5 into Cytoscape and activate the SyNDI application.

Install connecton to Galaxy

Open the Galaxy interface and generate a public API key. This option can be reached trough the top menu, select the menu ‘user’ and then the option ‘API keys’. Click ‘Generate a new key now’ and copy the newly created key into the clipboard buffer. In Cytoscape go to the ‘SyNDI – Gal’ tab and click the ‘Configure Galaxy Server’ button. Paste the public API key and enter the IP address of your Galaxy server instance. If you run Galaxy locally, please enter ‘127.0.0.1’.

Install ‘naive operon extend’

Press the ‘Load gene directions’ button and select ‘genedirections.tsv’ file from Additional file 5.

‘Naive operon extend’ operation

The operation can be executed by a single click on the ‘naive operon extend’ button.

‘Apply Meme2Fimo’ operation

Operation can be executed with the following steps.

- Press ‘Add selected genes to input’
- Press ‘run Meme2Fimo’.
- Press ‘Upload Fasta File With Upstream Regions’ and select file ‘upstream_1000_rsat.fasta’ from Additional file 5.
- Press ‘Upload Background File For Upstream Regions’ and select file ‘upstream_1000_rsat.bg’ from Additional file 5.

- Press ‘Upload Complete Genome Sequence’ and select file ‘H37Rv.fasta’ from Additional file 5.
- Press ‘Upload Background for Genome’ and select file ‘H37Rv.bg’ from Additional file 5.
- Press ‘Upload Annotation for Genome’ and select file ‘H37Rv_v2.gbk’ from Additional file 5.
- Press ‘Run’
- Go to Galaxy interface for the result
