# Supplementary material for: SyNDI: synchronous network data integration framework
Source: BMC Bioinformatics. 2018 Nov 6;19:403. doi: 10.1186/s12859-018-2426-5 (PMC6219086; doi:10.1186/s12859-018-2426-5)
Supplement: Supplementary file 70 [file 12859_2018_2426_MOESM7_ESM.zip › Additional_file_7/Constr_instructions.odt]

Step by step construction instructions – Scalability analysis

The purpose of this document is to describe step-step by step how we constructed a synchronous network visualization for scablity analysis.

We have tested these instructions on an ordinary desktop computer with the below metioned technical details.
Operating system: Ubuntu 16.04 LTS

Memory: 15,6 GB

Processor: IntelCore i7-3770 CP, 3.40GHz × 8

We constructed the SyncVis_scal_analysis.cys file by going through the below mentioned steps.

- To install SyncVis on our computer, we copied syncvis-1.0.1.jar to the $HOME_FOLDER/CytoscapeConfiguration/3/apps/installed folder. $HOME_FOLDER is the home folder on my comptuer.

- We opened Cytoscape as adminstrator by giving the following command:
sudo ./Cytoscape

- We imported each 11 networks from the Nets folder individually Cytoscape using the “ctrl + L” shortcut.

  - After each import, we re-named the network to the corresponding file name (e. g. maincor) by right mouse clicking on the network label on the network panel and the selecting “re-name”.

- Once we had imported all 11 networks, we imported selected nodes to these networks:

  - We clicked on the “Import Selected Genes From File” button under “SyncVis – Net” tab.

  - We got a filechooser on which we selected the "Biclusts/Bicluster_9.txt" file.

As a result all of these networks are visualized on SyncVis and the genes from the "Biclusts/Bicluster_9.txt" file were selected. A snapshot from this visualization is presented in Fig. 10 in the main text of the manuscript.
